# Construction of redundant communications to enhance safety against communication interruptions during robotic remote surgery

**DOI:** 10.1038/s41598-023-37730-9

**Published:** 2023-07-04

**Authors:** Hajime Morohashi, Kenichi Hakamada, Takahiro Kanno, Kotaro Tadano, Kenji Kawashima, Yoshiya Takahashi, Yuma Ebihara, Eiji Oki, Satoshi Hirano, Masaki Mori

**Affiliations:** 1 Committee for Promotion of Remote Surgery Implementation, Japan Surgical Society, Tokyo, Japan; 2 Department of Gastroenterological Surgery, Hirosaki University Graduate School of Medicine, Hirosaki, Japan; 3 Riverfield Inc., Tokyo, Japan; 4 Department of Information Physics and Computing School of Information Science and Technology, The University of Tokyo, Tokyo, Japan; 5 Department of Gastroenterological Surgery II, Hokkaido University Faculty of Medicine, Sapporo, Japan; 6 Department of Surgery and Science, Kyushu University, Fukuoka, Japan; 7 Tokai University School of Medicine, Isehara, Japan

**Keywords:** Electrical and electronic engineering, Surgical oncology

## Abstract

It is important to ensure the redundancy of communication during remote surgery. The purpose of this study is to construct a communication system that does not affect the operation in the event of a communication failure during telesurgery. The hospitals were connected by two commercial lines, a main line and a backup line, with redundant encoder interfaces. The fiber optic network was constructed using both guaranteed and best-effort lines. The surgical robot used was from Riverfield Inc. During the observation, a random shutdown and restoration process of either line was conducted repeatedly. First, the effects of communication interruption were investigated. Next, we performed a surgical task using an artificial organ model. Finally, 12 experienced surgeons performed operations on actual pigs. Most of the surgeons did not feel the effects of the line interruption and restoration on still and moving images, in artificial organ tasks, and in pig surgery. During all 16 surgeries, a total of 175-line switches were performed, and 15 abnormalities were detected by the surgeons. However, there were no abnormalities that coincided with the line switching. It was possible to construct a system in which communication interruptions would not affect the surgery.

## Introduction

Expectations are now rising for robot-assisted surgery to be more than just a way to perform surgeries, but also as a tool to bring new value to surgical care and society^1^. Specifically, remote medical treatment and remote surgery using information and communication devices are expected to be useful forms of medical treatment and therapy in areas where medical resources are scarce^2,3^. This expectation is because Japan is a country where surgical robots are becoming increasingly commonplace, and Japan has also focused much attention on the development of high-speed, high-capacity communication technologies such as optical fiber and 5G. As a result, an environment is being created in which robots in remote areas can be connected to each other to perform actual surgery.

Remote surgery was performed overseas for the first time more than 20 years ago between New York City and Strasbourg, France, about 7,000 km across the Atlantic Ocean^4,5^. However, telesurgery has not yet been put into practical use because there are many social, ethical, and economic issues that need to be addressed in addition to the technical issues that need to be verified to implement telesurgery in society. In February 2021, we connected Hirosaki City to Mutsu City, a distance of about 150 km, via a commercial line, using Riverfield's robotic system, which is still under development^6^. In August 2021, hinotori^TM^. (Medicaroid Corporation, Kobe, Japan) was successfully operated remotely over a distance of 2000 km using a Science Information NETwork (SINET)^7^. After verifying the communication delay and image quality at various bandwidths, we succeeded in establishing the communication environment required for the surgery.

In robotic telesurgery, the integrity and stability of communication is important. It is very important to ensure the redundancy of the communication even when the communication is interrupted due to unexpected equipment failure or other communication failure during the telesurgery. So far, there are no reports that discuss whether robotic telesurgery can be continued after communication interruption. The purpose of this study was to construct a robotic telesurgery system that uses two types of communication lines, and to investigate whether it is possible to construct a system that will enable an operation to continue after a communication interruption during actual telesurgery on a living being.

## Materials and methods

### Network connections

**Figure 1:**
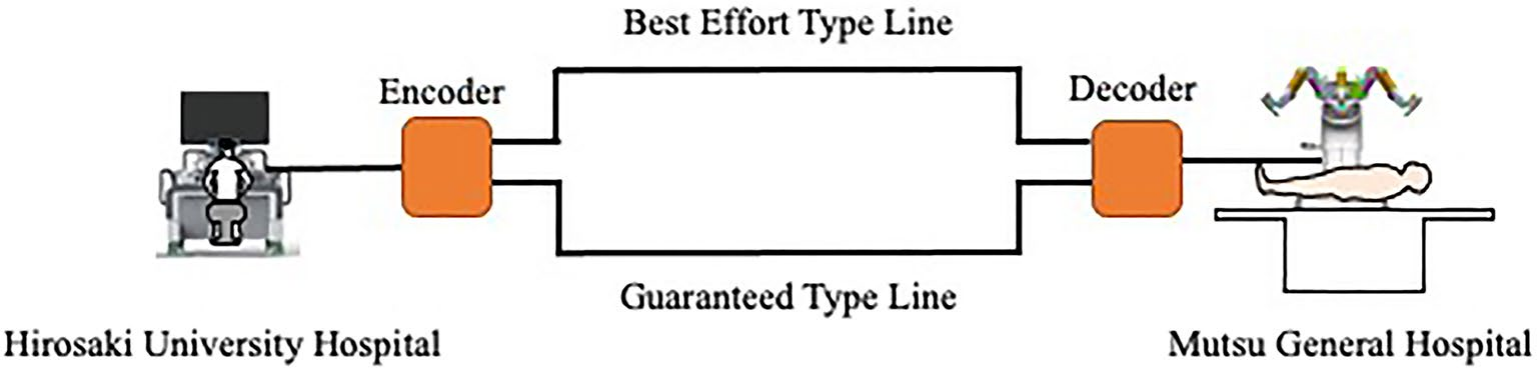
Network system, OUN: Optic network unit, CPE RT: Customer premises equipment remote terminal.

The Hirosaki University Hospital in Hirosaki City and Kitasato University School of Veterinary Medicine in Towada City, 100 km away, were connected through a fiber optic network. Hirosaki City and Towada City are located in the northernmost part of Japan’s Tohoku region. Both cities are known for poor transportation access and heavy snowfall in the winter. The fiber optic network was constructed using a guaranteed-type line (guaranteed bandwidth: 10 Mbps), which is an established private line not connected to the internet and best-effort lines established over an IP-VPN (Internet Protocol-Virtual Private Network). These lines were provided by NTT East (NIPPON TELEGRAPH AND TELEPHONE EAST CORPORATION). For compression and decompression of the communication information, we used the Zao-SH encoder and the Zao-View decoder from Soliton Systems K. K. Guaranteed lines and best-effort lines were directly connected to the encoder. The decoder was connected to the L3 network switch, which is the upper level of the network, and the L3 network switch was configured to make the two lines redundant (Fig. 1).

### Robot system

**Figure 2:**
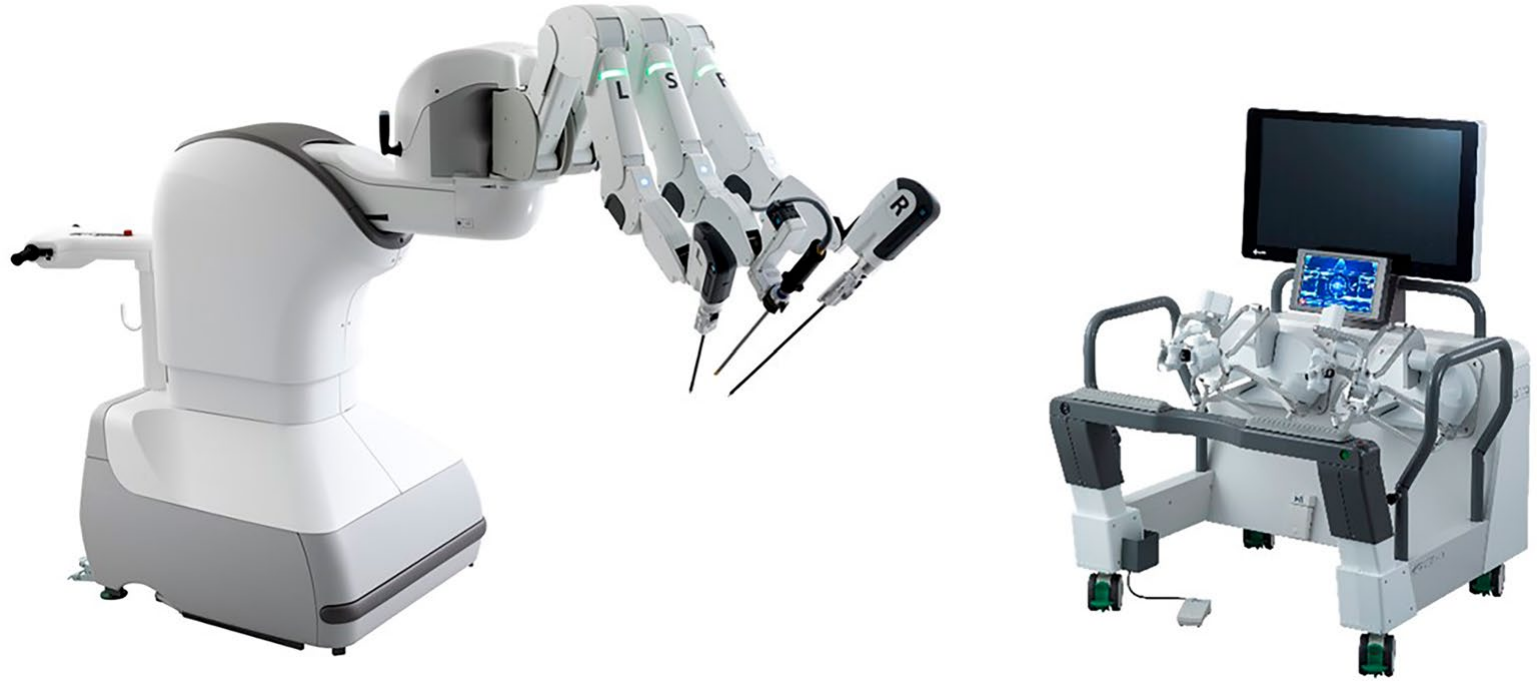
Riverfield robotic system, The photograph is a promotional material and may be different from the experimental apparatus.

The robot uses a pneumatically powered robotic system from Riverfield Inc (Tokyo, Japan)^8^(Fig. 2). The cockpit was installed at Hirosaki University and the robot was installed at Kitasato University.

### Evaluation methods of images by line switching

#### Subjective evaluation method

We developed two of our own question types, Question A and Question B. Question A was about whether or not the surgical technique was inhibited by the environment. Question B was about whether or not the surgery was actually possible (Supplementary Table 1).

#### Evaluation method of image quality

Image Quality Score A was used to analyze the type of image quality degradation that occurred due to changes in the communication environment and impact on the procedure. Image Quality Score A includes a scale for clarity, stereoscopic vision, completeness, continuity, and the effect on technique. The image quality rating scale is a 5-point scale, where a high score indicates that the image quality is not degraded and does not affect the procedure. The total score for each item was used for evaluation (Supplementary Table 2) Image Quality Score B was used to evaluate the degradation of image quality and its impact on the procedure by modifying Image Quality Score A. A high score indicates no degradation in image quality and no effect on the procedure (Supplementary Table 3).

#### Image evaluation for still and moving images

**Figure 3:**
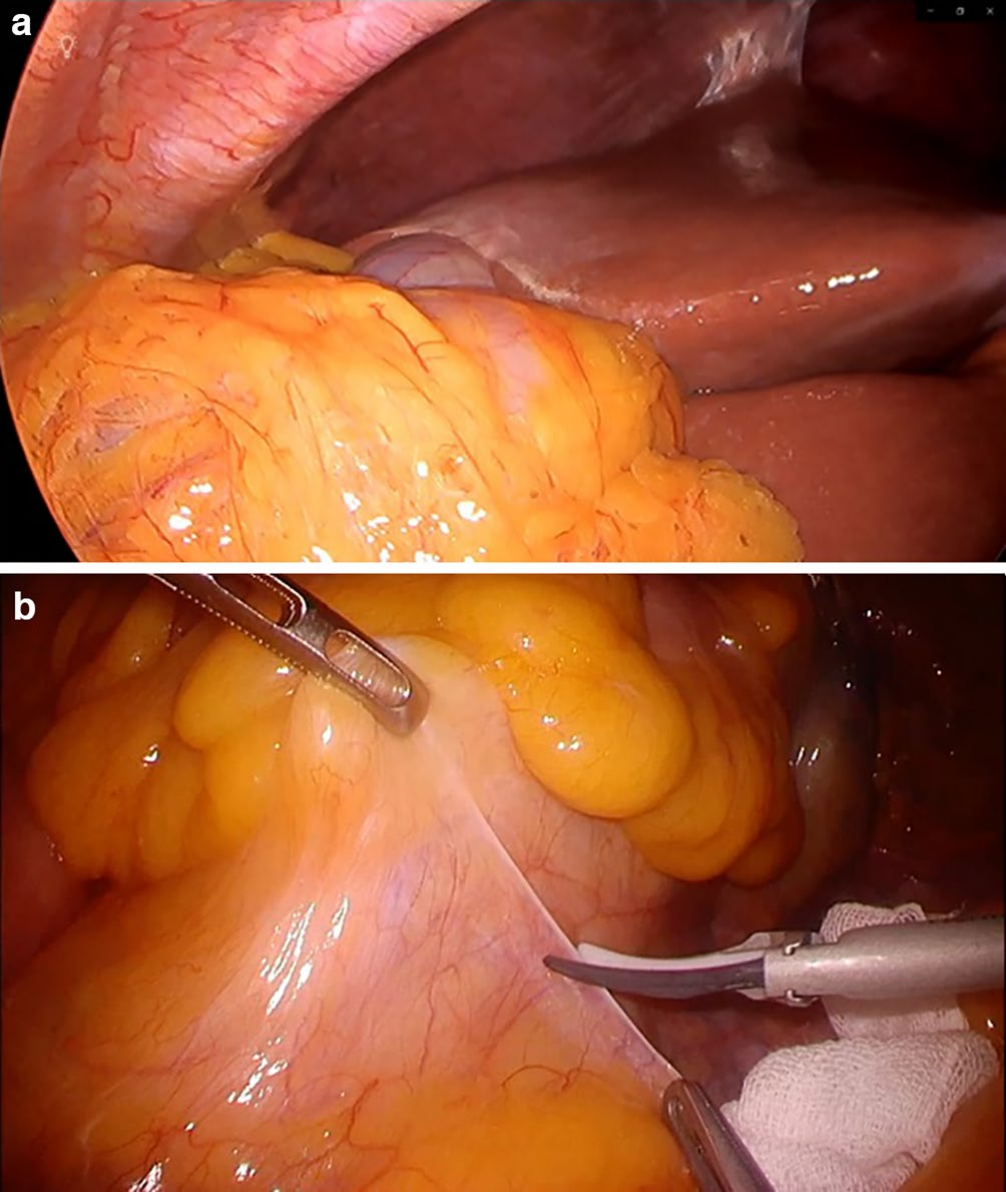
Still and moving images, (**a**) Still image, (**b**) Moving image: The figure shows a portion of the actual video used.

Still and moving images taken during the intraabdominal surgery were prepared on the monitor of the computer by the professionals in Towada City, and these images were captured by the camera installed on the Riverfield robot (Fig. 3a, b). Ten surgeons observed this image on a monitor in the cockpit on the Hirosaki City side for three minutes. During the observation, shutdown and restoration of either line were repeated randomly, and the changes to the image during the process were examined. One disconnection and one restoration took place during the execution of each task, for a total of 20 random line changes. A switch to turn on a lamp was placed at the surgeon's feet, and when there was a change, the surgeon activated the switch to report a change in the image. A subjective evaluation was conducted after the task was completed. Image quality was evaluated using Image Quality Scores A and B.

#### Task evaluation of artificial organ models

The six surgeons participate in the extensive tele-surgical experience. Each of them performed two different tasks using the cholecystectomy model and the intestinal suture model with and without line switching. Three disconnections and three restorations occurred during the execution of each task, for a total of 36 randomly executed line switches. The effect of communication interruptions on the continuity of robot operations during the task was examined.

**Figure 4:**
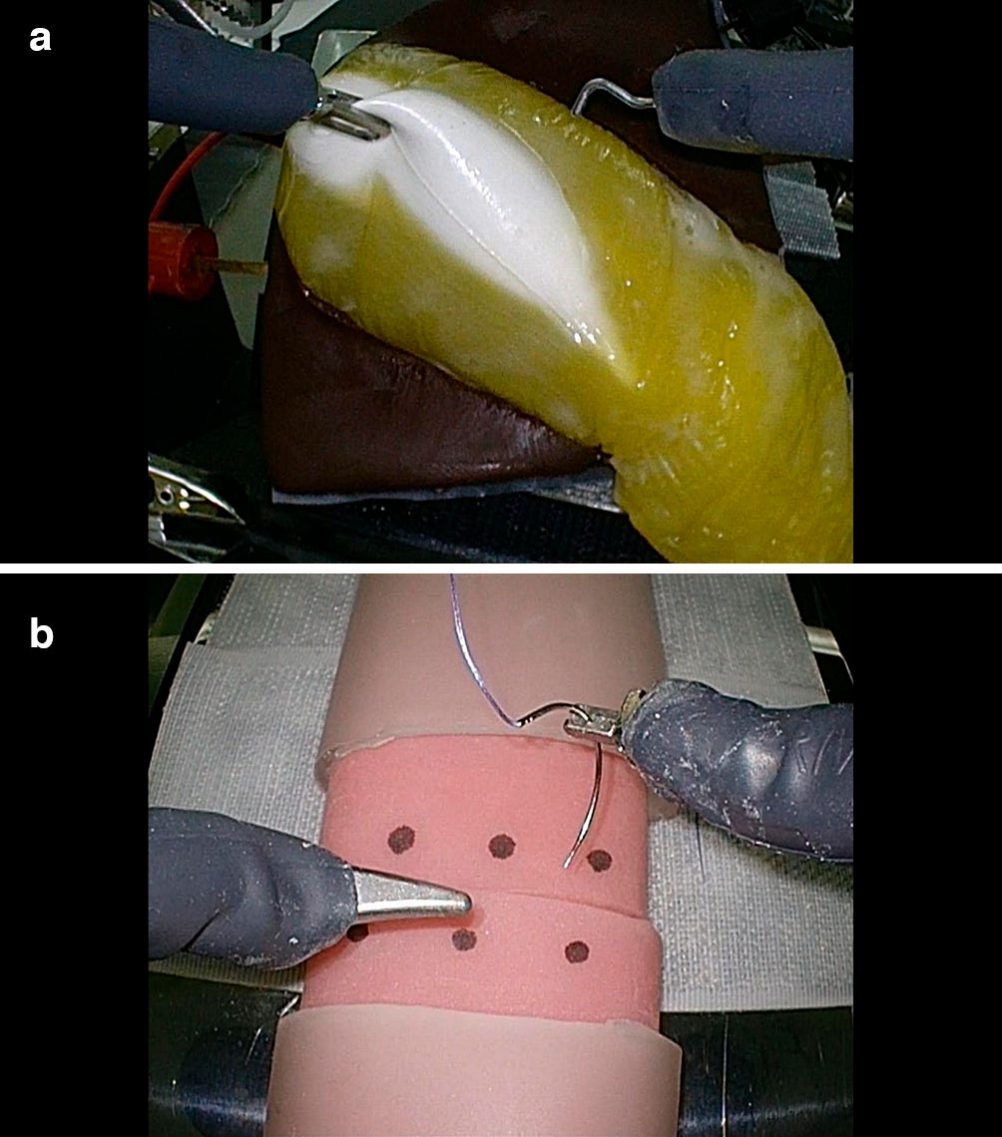
Artificial organ model, (**a**) Cholecystectomy model, (**b**) Intestinal suture model.

1. *Image evaluation*: When there was a change in the image during the task, the change in the image was reported by pressing down on the switch near the foot, and a subjective evaluation was conducted after the task was completed. The image quality was evaluated using Image Quality Scores A and B.
2. *Cholecystectomy model*: A cholecystectomy model made by FASOTEC Co. was used (Fig.4a). The gallbladder was removed from the gallbladder bed using monopolar electrocautery. The number of errors and the time required were evaluated by modifying the evaluation method reported by Sharker et al.^9^. The number of errors was scored by evaluating six items on a scale of YES = 1 point, NO = 0 points, and calculating the total score.
3. *Intestinal suture model:* For intestinal suturing, we used an intestinal model manufactured by AS ONE Corporation (Fig.4b). Three suture sites were marked at 5 mm intervals, and three single ligations were performed in one suture. The time from the start of suturing to the ligation and disconnection of the third suture were measured and evaluated, and the number of errors and time required were evaluated based on the evaluation method proposed by Goh et al.^10^. The number of errors was evaluated on six items, and the cumulative value of the number of errors for each was used as the score.

### Evaluation of surgery using pigs

**Figure 5:**
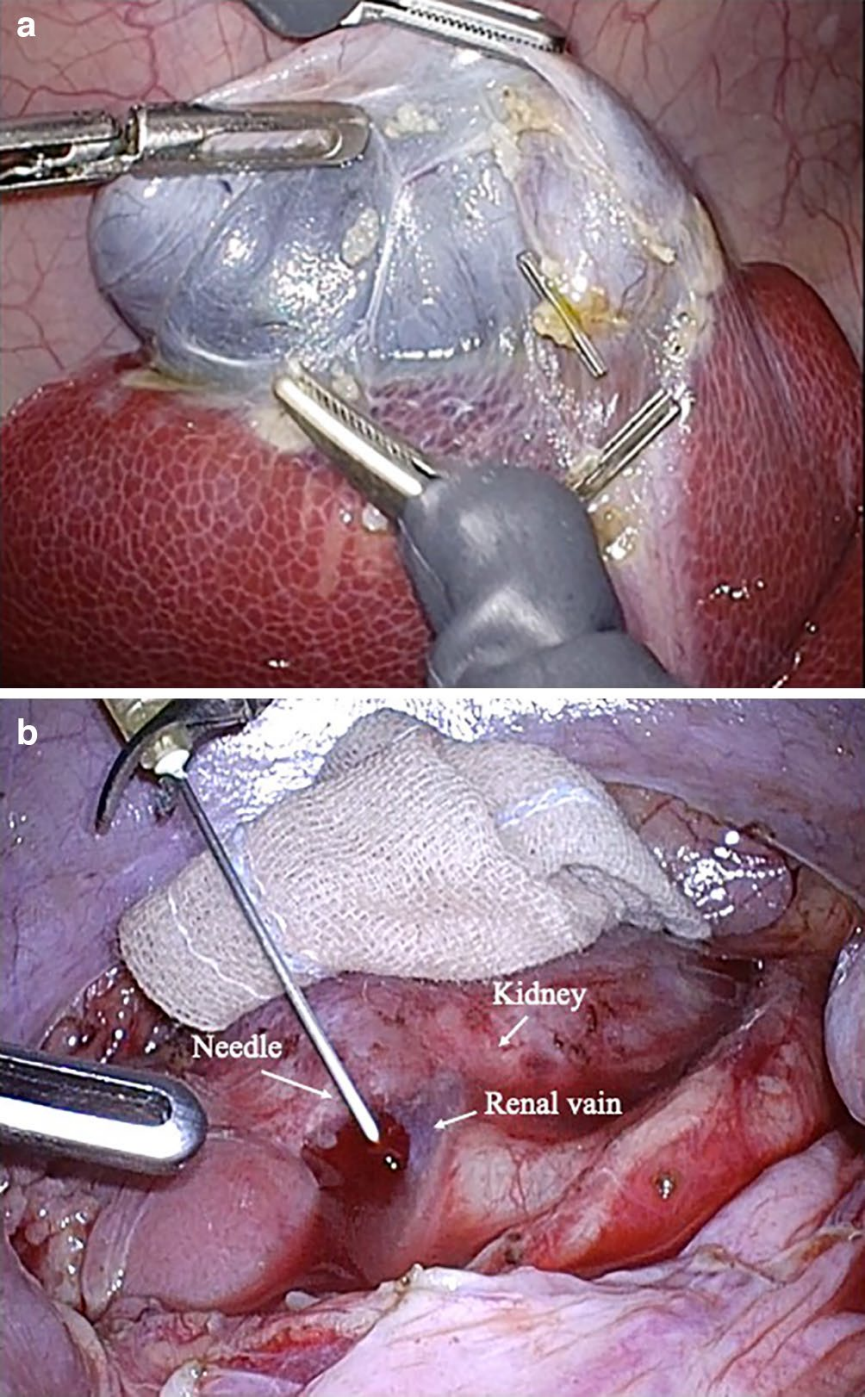
Remote robotic surgery using pigs, (**a**) Cholecystectomy of pigs by remote robotic surgery, (**b**) Renal vain puncture task using pigs.

A total of 16 experienced surgeons performed four different procedures, gastrectomy on four pigs (n = 4), rectal resection (n = 4), cholecystectomy (n = 4) (Fig. 5a), and nephrectomy (n = 4). Healthy pigs weighing between 26 and 29 kg were used, and the procedures were performed with a monopolar electrocautery scalpel as in standard human surgery. Anesthesia was induced with medetomidine hydrochloride 20 mg/kg + midazolam 0.2 mg/kg + butorphanol tartrate 0.2 mg/kg intramuscularly and maintained with isoflurane (2%). The pigs were euthanized with sodium pentobarbital 200 mg and KCL (20 mL) intravenously.

1. *Image evaluation*: Each surgeon was randomly assigned communication disconnection and restoration during remote surgery. Total 92 times disconnection and 83 times restoration were applied to all 16 surgeons. Whenever there was a change in the image during the task, the subjects pressed down on a switch at their feet to report the change in image, and a questionnaire was administered after the task was completed.
2. *Renal vain puncture task during communication interruption*: Four surgeons performed the task. A 20G needle was inserted into the renal vein to cause bleeding, and at the same time, one of the communication lines was cut off. The time from the puncture of the renal vein to the completion of compression hemostasis and the time from the grasping of the threaded needle to the completion of suture hemostasis were measured (Fig.5b).

#### Ethics

We conducted our study in accordance with ARRIVE guidelines (https://arriveguidelines.org). The use of pigs was reviewed and approved by Institutional Ethics Committee and the Animal Experimentation Committee of Hirosaki University for compliance with the Law Concerning the Protection and Management of Animals, the Basic Guidelines for Suffering of Animals in Experiments, and the Guidelines for Methods of Slaughtering Animals (M21022).　The entire study was approved by the aforementioned ethics committee. The surgeons are part of the study and informed consent was obtained from all of them. All methods were performed in accordance with the relevant guidelines and regulations.

## Results

### Effect of communication interruption on still and moving images

**Figure 6:**
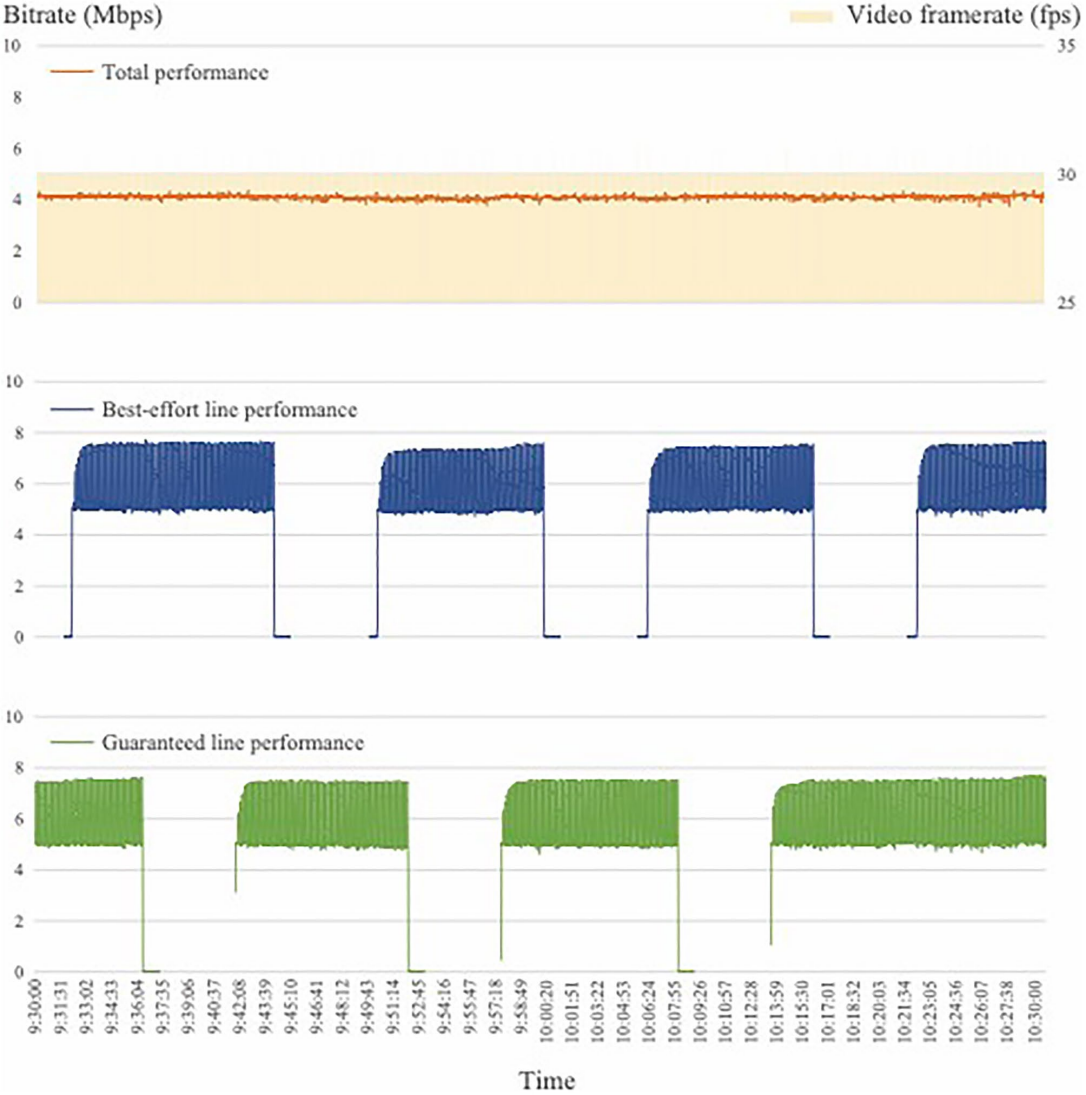
An example of the communication status during a certain time period during the remote surgery experiment.

Figure 6 shows an example of the communication status at a given period during the remote surgery experiment (Fig. 6). Switching between guaranteed and best-effort lines over approximately one hour did not affect the amount of information communication between them. The framerate and bitrate were stable at 30 fps and 4 Mbps, respectively. This communication stability was retained during all of the following tasks. For the still image and video tasks, the number of times the subject reacted to changes in the image was zero. In the subjective evaluation, the average score for Question A was 4.8, and for Question B was 4.9, with most subjects indicating that communication interruption had no effect on the still and moving images (Table 1).

**Table 1:** Subjective evaluation of still and moving image.

		Switching lines (N = 10)
Question A	Mean (Min–Max)	4.8 (3–5)
Question B	Mean (Min–Max)	4.9 (4–5)

### Impact on artificial organ model tasks

In the cholecystectomy model and the intestinal anastomosis task, one person each responded that they detected an abnormality with and without line switching. In Question A, the mean score for those who switched lines was 4.67, and the mean score for those who did not was 4.58, indicating no significant difference (*p* = 0.773). In Question B, the mean score of those who switched lines was 3.83, and the mean score of those who did not switch lines was 3.58, also with no significant difference (*p* = 0.536) (Table 2).

1. *Image quality score*: In Image Quality Score A, the mean score of those who switched lines was 21 and that of those who did not switch lines was 21, showing no significant difference (*p* = 0.918). In Image Quality Score B, the mean score for those who switched lines was 4.58 and 4.5 for those who did not switch lines, with no significant difference (*p* = 0.999) (Table 3). Specifically, anomalies in clarity, continuity, and operability were noted.
2. *Cholecystectomy model*: The mean time to complete the task for those who switched lines was 734.8 seconds, and for those who did not switch lines was 745.5 seconds (*p* = 0.936). The mean number of errors for those who performed line switching was 0.167 and for those who did not perform line switching was 0.67, with no significant difference (*p* = 0.233) (Table 4).
3. *Intestinal suture model*: The mean time to complete the task for those who switched lines was 506.0 seconds, and 505.7 seconds for those who did not (*p*=0.996). The mean number of errors for those who switched lines was 0.167 and for those who did not switch lines was 0.33, with no significant difference (*p* = 0.999) (Table 5).

**Table 2:** Subjective evaluation of artificial organ models.

		Switching lines (n = 12)	Not switching lines (n = 12)	*P*
Question A	Mean (Min–Max)	4.67 (4–5)	4.58 (4–5)	0.773
Question B	Mean (Min–Max)	3.83 (2–5)	3.58 (2–5)	0.536

**Table 3:** Image quality score of artificial organ models.

		Switching lines (n = 12)	Not switching lines (n = 12)	*P*
Image quality score A	Mean (Min–Max)	21 (16–25)	21 (18–25)	0.918
Image quality score B	Mean (Min–Max)	4.58 (3–5)	4.5 (3–5)	0.999

**Table 4:** Time and error in cholecystectomy model.

		Switching lines (n = 6)	Not switching lines (n = 6)	*P*
Time [sec]	Mean (Min–Max)	734.8 (517–1188)	745.5 (567–1102)	0.936
Error [number]	Mean (Min–Max)	0.167 (0–1)	0.67 (0–1)	0.233

### Effects of disconnection and restoration on pig surgery

1. *Gastrectomy, rectal resection, cholecystectomy, nephrectomy*: All surgical procedures were successfully performed by telerobotic manipulation. During all 16 surgeries, a total of 175-line switches were performed, and 15 abnormalities were detected by the surgeons. However, there were no abnormalities that coincided with the line switching. In addition, there were no image changes or operation abnormalities that would affect the progress of the surgery. In the subjective evaluation, the mean score for Question A was 4.53 and for Question B was 4.41 (Table 6).
2. *Hemostasis task*: The mean time taken from renal vein puncture to compression hemostasis was 8.88 seconds, and the time from grasping the threaded needle to completing suture hemostasis was 309.21 seconds. No particular effect of line interruption was observed, and remote suture hemostasis was successfully achieved in all cases thanks to line redundancy during communication switching (Table 7).

**Table 5:** Time and error in intestinal suture model.

		Switching lines (n = 6)	Not switching lines (n = 6)	*P*
Time [sec]	Mean (Min–Max)	506.0 (340–790)	505.7 (326–703)	0.996
Error [number]	Mean (Min–Max)	0.167 (0–1)	0.33 (0–2)	0.999

**Table 6:** Subjective evaluation of surgery using pigs.

		Switching lines (n = 17)
Question A	Mean (Min–Max)	4.53 (3–5)
Question B	Mean (Min–Max)	4.41 (3–5)

**Table 7:** Time to stop bleeding.

		Switching lines (N = 4)
Time from renal vain puncture to pressure hemostasis [sec]	Mean (Min–Max)	8.88 (4.30–12.09)
Time from needle hold to suture to stop bleeding [sec]	Mean (Min–Max)	309.21 (139.43–629.43)

## Discussion

By preparing two types of communication lines, we were able to continue a surgical procedure with almost no impact on robot operation even when one of the lines was interrupted. In other words, we were able to build a highly secure communication system for remote surgery. So far, telesurgery has been tackled in various fields by combining robotic technology and network communication technology^11–15^. In order to implement telesurgery in society, it is necessary to build a system that is prepared for any communication problem that may occur. The occurrence of communication problems can lead to the disruption of images and, consequently, the inability to operate the robot, which is a major obstacle to safe surgery. Therefore, it is necessary to have a line with redundancy in communication.

The problem of communication redundancy is not really an issue with the line, but rather a problem with the encoder and decoder. In this project, we built a system in which the interface of the encoder was made redundant; 100% of the video signals were sent out in duplicate on the main and backup lines, and the video signals received in duplicate by the decoder were output as a single image. This encoder-decoder has two modes: one is to transmit the same packet to multiple lines by duplicating it, and the other is to transmit the packet by dividing and distributing it. In the duplicate transmission mode, the encoder duplicates and transmits 100% of the robot signals and endoscope images to the two lines mentioned above. The decoder receives 200% of the packets and decodes 100% of the packets by extracting the packets with the fastest arrival speeds. The decoder also replicates 100% of the console's control signals on each of the two lines and sends them back to the encoder. Even if one of the two lines is slowed down, or one of the lines is artificially or intentionally disconnected at will, 100% of the control signals remain, so, the robot and the console stay connected. However, there is a difference of a few milliseconds in line delay between the two lines, and when the line with the shorter delay is disconnected, there is no communication until it switches over to the packet with the longer delay. We have customized the system to maintain the connection by having a buffer between the robot and the console. On the other hand, there is a mechanism on the encoding and decoding side to make the endoscope image appear uninterrupted. The encoders and decoders used in this study employ the Real-time Auto Speed Control based-on Waterway model (RASCOW™), which is a high compression technology that enables ultra-short delay video transmission and has been used in situations demanding minimum delay such as live broadcasting and remote control of construction equipment and automobiles.

In this study, to examine the changes in image quality due to communication interruptions, we disconnected and restored the line while the task was being performed and checked the number and timing of abnormal detection cues from the surgeon during that time. Abnormality detection by several subjects was confirmed. None of the subjects' detections coincided with the timing of the line disconnection and reconnection. The discrepancy in these results was attributed to the fact that many of the subjects were not familiar with the characteristics of the Riverfield robot, and thus mistakenly perceived slight shaking of the arm during robot operation or cloudy images caused by mist during surgery as abnormalities caused by line switching. This tendency appeared particularly strong during the more complex animal surgeries. Therefore, the subject's perception of abnormality was not due to an actual abnormality in image quality caused by communication switching but was thought to be due to a problem on the robot side and a change in the operating field situation. As shown in Fig. 4, the actual data communication information was stable, and there was no frame drop or packet loss.

As for the operability of the robot, in artificial organ models, robot operability continued due to the safeguard of communication switching and there were no problems that would affect the surgery. Similarly, in biological surgery, robot operability was maintained and there was the line switching had no effect that would compromise the surgery. In this study, we attempted to perform a hemostasis task under the conditions of artificial communication interruption, and all four subjects were able to apply accurate compression to the hemostasis point and complete suture hemostasis without being affected by the communication problem. It is often reported that it is desirable to keep the delay time below 100 ms in standard robotic surgery^16,17^. The delay time of this system was confirmed to be less than 100 ms, in advance^6^. In this experiment, there was no delay due to communication switching that would interfere with surgery. It was confirmed that the security of communication was maintained even when communication problems occurred. In general, the stronger the line, the more stable the communication and the more stable the robot operation will be, but the higher the communication fees will be. In telesurgery, it is necessary to select a communication network based on the premise of sufficient communication quality (securing bandwidth) and communication security, but also considering economic feasibility.

The number of robotic surgeries in general surgery in particular has been increasing as robotic surgeries have become more common in general procedures such as hernia surgery, as opposed to cancer surgery^18^. In the future, the need for robotic surgery is expected to expand more and more, both in urban and rural areas. In addition to the da Vinci Surgical System® by Intuitive Surgical, which is currently the mainstream system in the U.S., new robotic systems have been introduced in South Korea, the U.K., Germany, and other countries, and robots under development are scheduled to enter the market in the future^19^. It is expected that the oligopoly of robot sales will gradually improve as price competition arises, and that, like many electrical appliances and cars, robots will become more powerful and less expensive as they become more widely used. With the spread and generalization of robots, the role of robotic telesurgery will increase for the purposes of equal access to medical care and the education of young surgeons. For the global practical application of telesurgery, it is very important to ensure the redundancy of communication in order to strengthen the security of communication.

## Limitations

Due to the limited validation time, the number of subjects and the number of pigs used in the experiment were small. Many of the subjects were not familiar with the Riverfield robot. We were also unable to study the economics of communication in detail. There was insufficient consideration of the required bandwidth to improve the image quality required for the communication lines or the required bandwidth suitable for robot control.

## Conclusions

With the telerobot system constructed in this study, there was almost no effect on the images due to communication interruption during tasks using both non-living and living bodies. By providing two communication lines, it was possible to construct a system in which communication interruptions during robotic telesurgery would not affect the images during the surgery or the continuity of the robot operation.

## Supplementary Information

Supplementary Information.

## Data Availability

The data that support the findings of this study are available from the corresponding author [K. H.], upon reasonable request.
