# Supplementary material for: Construction of redundant communications to enhance safety against communication interruptions during robotic remote surgery
Source: Sci Rep. 2023 Jul 4;13:10831. doi: 10.1038/s41598-023-37730-9 (PMC10319872; doi:10.1038/s41598-023-37730-9)
Supplement: Supplementary file 1 — Supplementary Information. [file 41598_2023_37730_MOESM1_ESM.docx]

Supplementary Table 1: Each of the five questions about surgical environment was evaluated on a five-point scale

| Question A: Has this surgical environment inhibited with your surgical technique? | | | | |
| --- | --- | --- | --- | --- |
| I was inhibited　　　　　　　　　　 　I was not inhibited | | | | |
| 1 | 2 | 3 | 4 | 5 |

| Question B: Can you perform actual surgery in this surgical environment? | | | | |
| --- | --- | --- | --- | --- |
| I can’t　　　　　　　　　 　I can | | | | |
| 1 | 2 | 3 | 4 | 5 |

Supplementary Table 2: Each of the five questions about image quality was evaluated on a five-point scale

**Image Quality Score A**

**Please answer the following questions about the surgical images of the robotic surgery you performed.**

On a scale of 1 to 5, with 5 being considered satisfactory for performing the procedure and 0 being considered completely unsuitable for performing the procedure, please rate the procedure.

**1. Clarity：How clear were the images from the robotic surgery?**

| It's not clear at all　　　　　　　　　　　　　　　　　　　　　　　　　　　Very clear | | | | |
| --- | --- | --- | --- | --- |
| 1 | 2 | 3 | 4 | 5 |

**2. Stereoscopic vision：Were the images of the robotic surgery in stereoscopic vision?**

| Was not stereoscopic vision at all　　　　　　　　　　　　　　Very stereoscopic vision | | | | |
| --- | --- | --- | --- | --- |
| 1 | 2 | 3 | 4 | 5 |

**3. Completeness：Was the robotic surgery screen complete?**

| Incomplete　　　　　　　　　　　　　　　　　　　　　　　　　　　　　　Complete | | | | |
| --- | --- | --- | --- | --- |
| 1 | 2 | 3 | 4 | 5 |

**4. Continuity：Were the robotic surgery screens continuous?**

| There's no continuity at all　　　　　　　　　　　　　　　　It's completely continuous | | | | |
| --- | --- | --- | --- | --- |
| 1 | 2 | 3 | 4 | 5 |

**5. Impact on the procedure：Were you able to perform the procedure with the images from this robotic surgery?**

| Could not perform at all　　　　　　　　　　　　　　　　　　Could be done perfectly | | | | |
| --- | --- | --- | --- | --- |
| 1 | 2 | 3 | 4 | 5 |

Total score:

Supplementary Table 3: The degradation of image quality was evaluated using a five-step evaluation scale

**Image Quality Score B**

**Please rate the quality of the surgical images of the robotic surgery performed this time on a scale of 1-5 according to the following criteria, and a continuous rating on a number line from 1-5.**

**5: I don't feel any degradation in image quality.**

**4: Image quality degradation is present, but not bothersome.**

**3: Image quality is degraded, but it does not interfere with surgery.**

**2: Image quality is degraded, but it does not interfere with surgery.**

**1: Image quality is degraded and I cannot perform surgery.**

**1)　Circle one of the numbers from 1-5.**

| 1 | 2 | 3 | 4 | 5 |
| --- | --- | --- | --- | --- |

**2)** **Mark on the number line from 1-5.**

**1　　　　　　　　　　　　　　　　　　　　5**
